# Circulating Survivin Protein Levels in Lung Cancer Patients Treated With Platinum-Based Chemotherapy

**DOI:** 10.3389/pore.2021.631969

**Published:** 2021-04-30

**Authors:** Rita Puskas, Andras Bikov, Peter Horvath, Zsofia Lazar, Laszlo Kunos, Reka Nagy, Gabriella Pinter, Gabriella Galffy

**Affiliations:** ^1^ Department of Pulmonology, Faculty of Medicine, Semmelweis University, Budapest, Hungary; ^2^ Törökbálint Pulmonology Hospital, Törökbálint, Hungary; ^3^ Manchester University NHS Foundation Trust (MFT), Manchester, United Kingdom; ^4^ Semmelweis University, Budapest, Hungary; ^5^ Department of Thoracic Surgery, Faculty of Medicine, Semmelweis University, Budapest, Hungary

**Keywords:** survivin, lung cancer, ELISA, disease progression, biomarker

## Abstract

The survivin protein contributes to the development and progression of tumors. Protein expression and mRNA levels correlate with clinicopathological parameters and survival of cancer patients. Our purpose was to evaluate whether circulating survivin levels have any diagnostic or predictive value in lung cancer. 118 patients with advanced stage lung cancer participated in our study. 53 suffered from adenocarcinoma (ADC), 33 from squamous cell carcinoma (SqCC), and 32 from small cell lung cancer (SCLC). We also enrolled 21 control subjects. Blood samples were collected before and after two cycles of chemotherapy. We measured survivin concentrations with ELISA. Non-parametric tests were used for analysis. We did not find significant difference in survivin levels between patients and control subjects (17.19/0–829.74/vs. 49.13/0–165.92/pg/ml; *p* = 0.07). We found lower survivin concentrations in patients with SqCC (0/0–171.24/pg/ml) than in those with ADC (24.94/0–626.46 pg/ml) and SCLC (45.51/0–829.74/pg/ml) (ADC vs. SqCC *p* < 0.0001, ADC vs. SCLC *p* = 0.0405, SqCC vs. SCLC *p* < 0.0001). Survivin levels were higher in stage IV patients than in patients without distant metastases (*p* = 0.0061), and concentrations were progressively higher with increasing number of metastatic organ sites (*p* = 0.04). We observed a decrease in survivin levels in ADC patients after platinum plus pemetrexed chemotherapy (26.22/0–626.46/pg/ml before vs. 0/0–114.36/pg/ml after; *p* = 0.01). Neither progression-free nor overall survival correlated with survivin levels at baseline. Our data imply that survivin may be involved in the development of metastases and it might be used as a biomarker of disease progression. However, circulating survivin concentrations do not predict survival of patients with lung cancer.

## Introduction

Survivin (also called Baculoviral Inhibitor of Apoptosis Repeat-Containing 5, BIRC-5) is a member of the Inhibitor of Apoptosis Proteins (IAP) family [1]. Survivin has a key role in cell division, participates in the creation of the mitotic spindle and appears to contribute to the preservation of the stem cell state [2–4]. Its level changes during the cell cycle: the highest being during the G2/M transition, and the lowest being in the G1 phase.

Survivin plays an essential role in tumorigeneses in multiple ways: it inhibits caspase-3, -7 and -9 proteins, and blocks p53 as a caspase-independent pathway of apoptosis [5–7]. Survivin also regulates cell motility via the Akt - *α*-5-integrin pathway, and promotes the development of metastases [8]. Fernández et al. [9] described the process how survivin increases VEGF production and improves angiogenesis in tumor cells via PI3K/Akt enhanced *β*-catenin-Tcf/Lef-dependent transcription.

The pro-oncogenic effect of survivin is observed in many types of tumors including lung cancer. Multiple studies confirmed that in histological samples from patients with lung cancer, both survivin mRNA and protein expression are higher than in healthy lung tissue [10–12] Yie et al. [13] found the same results by measuring survivin mRNA in circulating tumor cells from peripheral blood samples. Furthermore, survivin protein expression assessed by immunohistochemistry in lung cancer tissue specimens correlates with the grade of differentiation, the tumor stage and the presence of lymph node metastases [10]. However, there are controversial results about the relationship between survivin expression and histological types [10, 11, 14, 15].

In surgically resected lung adenocarcinomas higher survivin protein expression indicated shorter disease-free and overall survival [16]. Beyond the correlation with tumor stage, primary tumor size and nodal state, elevated survivin mRNA levels in circulating tumor cells are related to increased relapse rate and shorter overall survival [13]. In blood samples of patients with operable non-small cell lung cancer (NSCLC), preoperative survivin mRNA positivity correlated with a worse prognosis, decreased survivin mRNA levels were measured after surgical resection, and still positive postoperative survivin mRNA indicated more frequent tumor recurrence and shorter disease-free and overall survival [17]. Measuring circulating tumor cell mRNA in peripheral blood samples of patients receiving chemotherapy, researchers found that progression-free and overall survival were worse in those patients whose survivin levels were higher before, after one, and after 3 cycles of chemotherapy [18]. Previously, only a few studies investigated blood survivin protein levels in lung cancer and did not find significantly different concentrations compared to controls [19, 20]. However, these studies were limited in the number of participants and both included only NSCLC.

Therefore, the aim of our study was to evaluate the relationship between circulating survivin protein levels and clinicopathological features, the effect of chemotherapy and the survival of patients suffering from advanced stage lung cancer.

## Patients and Methods

### Subjects

118 patients suffering from advanced stage lung cancer participated in our study. The diagnosis was based on bronchial or transthoracic histological or cytological sampling. Fifty-three patients were diagnosed with adenocarcinoma (ADC), 33 with squamous cell carcinoma (SqCC) and 32 with small cell lung cancer (SCLC). Twenty-five patients with ADC received platinum-based chemotherapy with bevacizumab, 25 received platinum plus pemetrexed. The other 3 patients received immunotherapy or platinum plus gemcitabine or best supportive care. Twenty patients with SqCC received platinum plus gemcitabine, and 24 patients with SCLC received platinum plus etoposide. Twenty-four cancer-free volunteers served as controls. We recorded tumor stage, the location of metastases, lung function results, the applied anticancer therapy, smoking habits and cigarette pack years. If a subject quit smoking at least 6 months before the diagnosis we considered them ex-smoker. Progression free and overall survival related to first line therapy were also documented, we followed the patients from December 2015 until March 2019. Median follow-up time was 10 months (0–42). The characteristics of our study subjects are summarized in Table 1.

**TABLE 1:** Patient characteristics.

	Adenocarcinoma (*N* = 53)	Squamous cell carcinoma (*N* = 33)	Small cell lung carcinoma (*N* = 32)	Control group (*N* = 24)	*p* value
Age, years, mean ± SD	63 ± 8	67 ± 8	63 ± 8	58 ± 9	0.002
Gender, N					0.03
Male	25	24	19	9	
Female	28	9	13	15	
Smoking, N					0.03
Current	26	20	20	20	
Previous	11	10	5	1	
Never	3	1	1	3	
Unknown	13	2	6	0	
Pack-years, mean ± SD	35.94 ± 24.96	45.67 ± 24.52	36.05 ± 24.79	35.55 ± 18.87	0.27
COPD, N					0.0004
yes^a^	21	20	11	7	
No	26	11	17	6	
Not assessed	6	2	4	11	
Tumor stage^b^					0.002
IIIA (inoperable)	0	3	2		
IIIB	10	15	10		
IV	39	8	18		
Unknown	4	7	2		
Number of metastatic sites					0.001
0	11	23	13		
1	24	6	12		
>1	13	2	6		
Unknown	5	2	1		
Progression-free survival (months, median/95% CI/)	5/4–6/	5/3–8/	7/6–9/		0.01
Overall survival (months, median/95% CI/)	10/7–16/	10.5/5–16/	8/6–15/		0.04

^a^ postbronchodilator FEV_1_/FVC < 0.7.

^b^ 8th Edition of the TNM Classification for Lung *Cancer*–IASLC.

The study was approved by the local Ethics Committee (Semmelweis University TUKEB 238/2015), and all subjects signed an informed consent.

### Sample Collection and ELISA Measurement

We collected venous blood samples before the first cycle of systemic chemotherapy, and after 2 cycles. EDTA-treated blood samples were centrifuged within 2 h at 1500 RPM for 10 min at 4°C. Immediately following centrifugation, plasma was separated into 250 µL aliquots which were stored at −80°C until analysis. Samples were thawed just before the ELISA measurements. To measure survivin protein levels we used a commercially available ELISA-kit (Bio-Techne R&D Systems Quantikine ELISA Human Survivin Immunoassay). The detection limit of the assay is 9.96 pg/ml, intra-assay variability is 17.38 ± 10.07%.

### Statistical Analysis

Statistical analyses were performed with Statistica 12 (StatSoft, Inc. Tulsa, OK, US) and Graph Pad Prism 5.0 (GraphPad Software, San Diego, CA, US). Normality of data was assessed with Kolmogorov-Smirnov test. Categorical and continuous variables as well as survivin concentrations were compared between the groups with Chi-squares, ANOVA and Kruskal-Wallis tests. Wilcoxon test was used to compare survivin levels before and after chemotherapy in ADC and SCLC groups. Correlations between survivin levels and clinical variables were assessed with Spearman test. The effect of survivin on progression free and overall survival was investigated with Cox regression. *p*-value <0.05 was considered significant.

## Results

### Survivin Levels in the Histological Groups Before Chemotherapy

**FIGURE 1:**
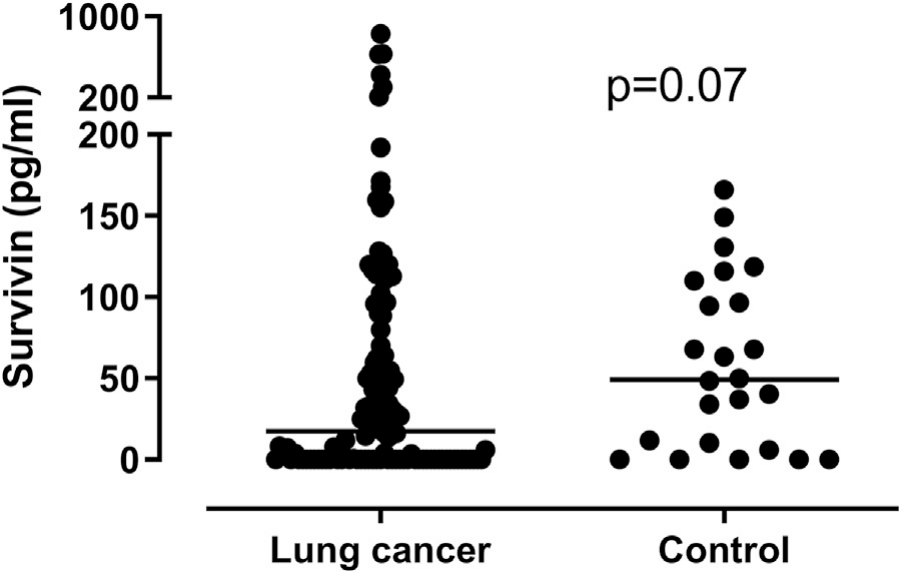
Plasma survivin levels in lung cancer patients and control subjects. There is no significant difference in survivin levels between patients and control subjects (*p* = 0.07). Horizontal lines show group median values.

**FIGURE 2:**
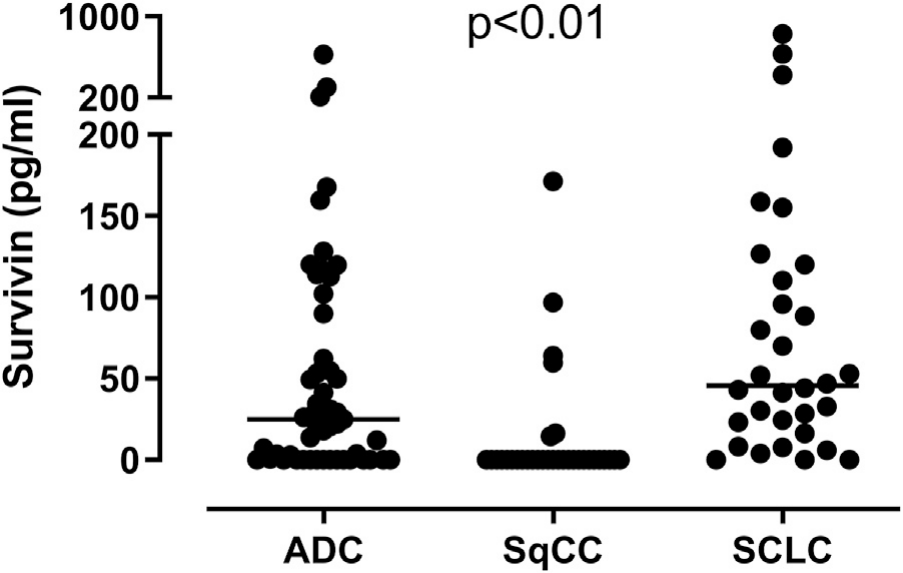
Plasma survivin levels in adenocarcinoma (ADC), squamous cell carcinoma (SqCC) and small cell lung cancer (SCLC). Survivin concentrations are lower in patients with SqCC than in those with ADC and SCLC (ADC vs. SqCC *p* < 0.0001, ADC vs. SCLC *p* = 0.0405, SqCC vs. SCLC *p* < 0.0001). Horizontal lines show group median values.

There was no significant difference in plasma survivin levels between patients with lung cancer and control subjects (17.19/0–829.74/vs. 49.13/0–165.92/pg/ml; *p* = 0.07). (Figure 1). Assessing separately the NSCLC and SCLC groups, we found significantly lower survivin levels in patients with NSCLC than in those with SCLC or in control subjects (NSCLC vs. SCLC *p* = 0.0053, NSCLC vs. controls *p* = 0.0004), but there was no significant difference between patients with SCLC and control subjects (SCLC vs. controls *p* = 0.3344). Comparing the histological groups we found significantly lower circulating survivin levels in patients with SqCC (0/0–171.24/pg/ml) than in patients with ADC (24.94/0–626.46 pg/ml) or SCLC (45.51/0–829.74/pg/ml) (ADC vs. SqCC *p* < 0.0001, ADC vs. SCLC *p* = 0.0405, SqCC vs. SCLC *p* < 0.0001). (Figure 2).

### Survivin and Clinical Factors

**FIGURE 3:**
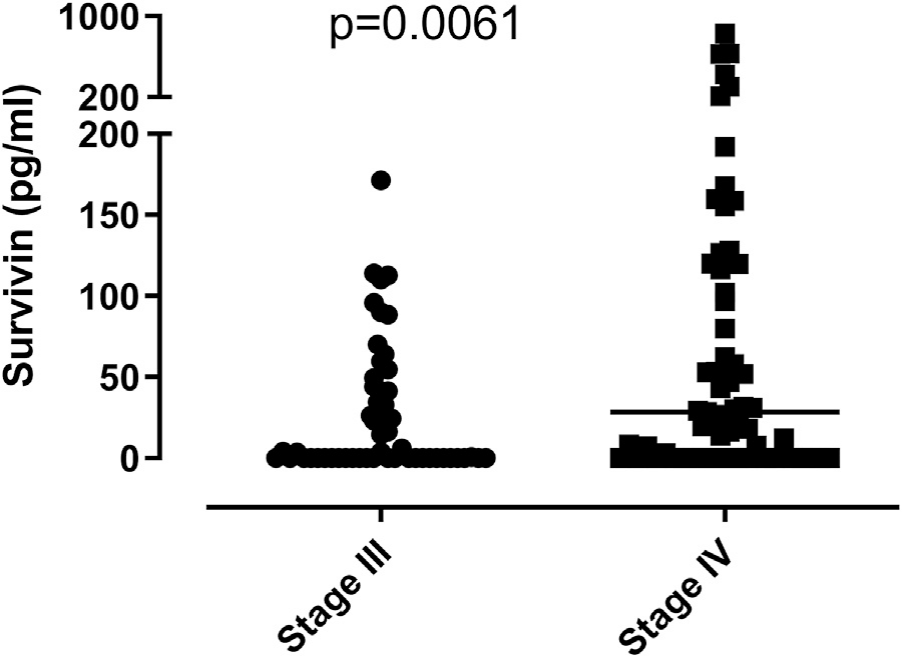
Plasma survivin levels and tumor stages. Survivin levels are higher in patients with stage IV lung cancer than in patients with stage III disease (*p* = 0.0061). Horizontal lines show group median values.

**FIGURE 4:**
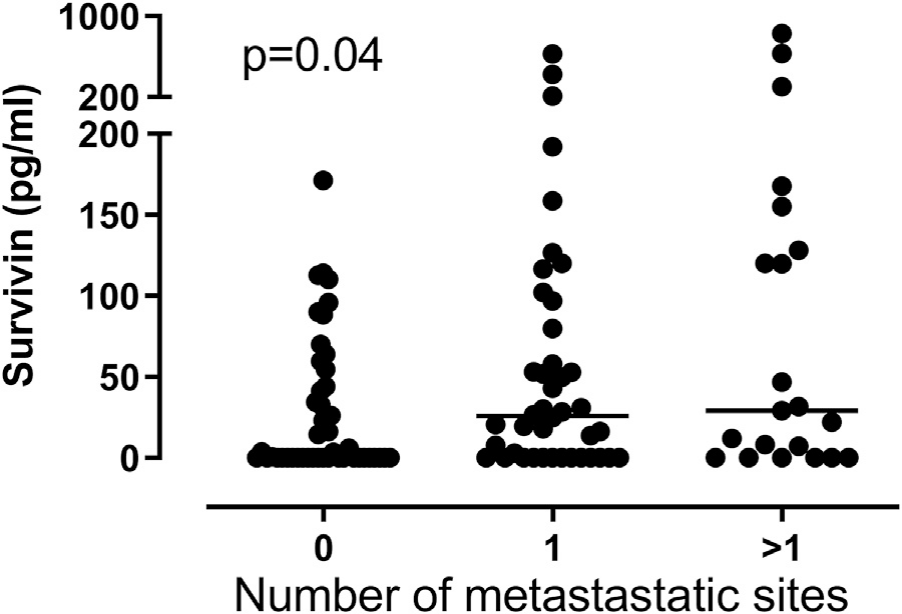
The relationship between plasma survivin levels and the number of metastatic sites. Survivin levels are progressively higher with increasing number of metastatic organ sites (*p* = 0.04). Horizontal lines show group median values.

Survivin concentrations were significantly higher in patients with stage IV disease (28.39/0–829.7/pg/ml) than in patients without distant metastases (0.51/0–171.2/pg/ml), *p* = 0.0061 (Figure 3). We found a direct relationship between survivin levels and the number of metastatic organ sites (*p* = 0.04) (Figure 4). We did not find significant difference between survivin levels in previous and current smokers (*p* = 0.52), and survivin concentrations did not correlate with the number of cigarettes smoked either (*p* = 0.72). Circulating survivin concentrations tended to be lower in patients with COPD (0/0–830 pg/ml) compared to those without (23/0–630/pg/ml, *p* = 0.06).

### Survivin Levels After Chemotherapy

**FIGURE 5:**
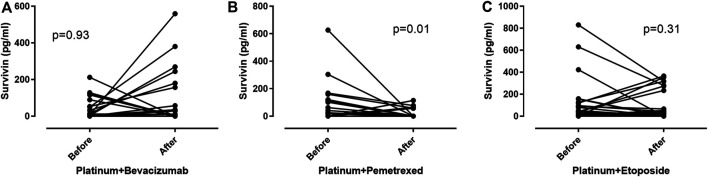
The effect of chemotherapy on survivin levels. **(A)**: There is no significant changes in plasma survivin levels in patients with adenocarcinoma due to platinum plus bevacizumab treatment (*p* = 0.93). **(B)**: Plasma survivin levels significantly decrease in patients with adenocarcinoma due to platinum plus pemetrexed treatment (*p* = 0.01). **(C)**: There is no significant changes in plasma survivin levels in patients with small cell lung cancer due to platinum plus etoposide treatment (*p* = 0.31).

To investigate the change of circulating survivin protein levels in reference to cytostatic treatment, we compared survivin levels before and after two cycles of chemotherapy. We observed a significant decrease in survivin levels after platinum plus pemetrexed combination in patients with lung adenocarcinoma (26.22/0–626.46/pg/ml before vs. 0/0–114.36/pg/ml after chemotherapy, *p* = 0.01), but there was no significant change in either bevacizumab-combined treatment in ADC or in small cell lung cancer (Figure 5).

### Survivin and Patient Survival

Our objective was to evaluate whether circulating survivin levels have prognostic or predictive values in lung cancer. We found no significant correlation between initial survivin levels and progression-free survival (*p* = 0.55) or overall survival (*p* = 0.89) in the entire tumorous group. Dividing patients to non-small cell and small cell lung cancer groups, the Cox-regression analysis did not show significant correlation between survivin concentrations and survival parameters (*p* = 0.60 for progression-free survival and *p* = 0.16 for overall survival in NSCLC; *p* = 0.06 for PFS and *p* = 0.07 for OS in SCLC), although, in the case of SCLC there was a tendency for higher survivin levels associating with worse outcomes. Since survivin has a role in angiogenesis we investigated separately the group receiving chemotherapy combined with VEGF-inhibitor bevacizumab, but again, we did not find correlation between survivin levels at baseline and survival parameters (*p* = 0.56 for progression-free survival and *p* = 0.42 for overall survival).

## Discussion

Despite the rapid development of systemic anticancer treatments, the survival of advanced stage lung cancer is still very poor. Suitable biomarkers which could help early diagnosis or therapeutic decision making may improve the outcomes of these patients. Therefore, intensive research on new biomarkers is essential.

In the present study we measured circulating survivin protein concentrations in plasma samples of advanced stage lung cancer patients; and assessed whether it could be a useful biomarker in the clinical routine. To our knowledge, this has been the largest study to test these parameters. Of importance, we enrolled patients with NSCLC and also with SCLC, to broaden the clinical interpretation of the study. Major limitations of our study are the relative low case numbers in certain histological subgroups such as in SCLC, raising difficulties in interpretation of some results, and the lack of post-therapy samples in patients with SqCC. Further limitation is that we collected plasma samples from the patients only once or twice (before and after chemotherapy), however a more frequent sample collection during the course of the disease could have provided more information about the role of survivin in disease progression.

Comparing plasma survivin concentrations in different histological groups, we observed that survivin levels were lower in patients with SqCC than with ADC or SCLC. The results of the previous studies, using other methods for survivin quantification than plasma measurements, are controversial in this aspect: some researchers could not confirm an association with the histological type [10], while others reported higher survivin expression in squamous cell carcinoma than in adenocarcinoma [11]. On the contrary, Porebska et al. [15] found lower survivin expressions in tissue samples of patients with squamous cell carcinoma, than those with lung adenocarcinoma, which is in line with our findings. Lower circulating survivin levels in squamous cell carcinoma might be in connection with the relatively moderate tendency to invasion and metastatization compared to other histological types.

Several studies assessed the correlation between survivin levels–measured in different ways–and the extension of lung cancer. Most [10, 11, 18], but not all [12] studies reported higher survivin expression in patients with advanced stages (III-IV). In our study we enrolled only patients with inoperable, advanced stage (mostly stage III B or IV) lung cancer. Plasma survivin concentrations were significantly higher in patients with distant metastases (Figure 4). When we divided the patients according to the number of metastatic organ sites (no distant metastasis, metastasis in one or in more than one organ), we found significant correlation between survivin levels and the extent of the disease.

The number of metastatic organ sites is considered as a predictor of survival in non-small cell lung cancer. The prominent difference between the survival rates of oligometastatic disease and tumor with multiple distant metastases created the need for distinguishing M1b and M1c diseases in the 8th edition of the lung cancer TNM classification system [21]. An increased number of metastatic organ sites correlates with lower survival rates [22, 23]. Du et al. [18] investigated survivin mRNA in circulating tumor cells in the peripheral blood of patients with advanced stage non-small cell lung cancer. They found that the presence of survivin mRNA in these cells is associated with the number of metastatic sites. Our results are in line with these previous findings and support the role of survivin in metastasis formation and invasion into distant organs.

Former studies, which measured survivin mRNA levels in circulating tumor cells in patients suffering from lung cancer, demonstrated that survivin levels change after chemotherapy. The decrease in survivin levels after cytostatic treatment was associated with a better outcome [18, 24]. Derin et al. [25] investigated the concentrations of serum survivin protein by ELISA technique in advanced stage non-small cell lung cancer patients before and after cytostatic treatment, and they demonstrated a significant decrease in survivin levels in the chemoresponsive group. In line with our results, they did not find significant difference between patients with lung cancer and control subjects, and the initial survivin levels did not correlate with the survival.

In our present study we compared plasma survivin levels before and after two cycles of chemotherapy. A decreased level of the protein was present only in the adenocarcinoma group treated with the platinum plus pemetrexed. The background of the isolated changes induced by the platinum plus pemetrexed therapy has not been clarified yet. Folate receptor *α* (FR-*α*) is one of the receptors, which is needed for the intracellular uptake of folic acid and also pemetrexed by cancer cells. FR-*α* also has a role independently from the nucleotide synthesis in promoting tumor cell proliferation and inhibiting apoptosis by activating ERK signaling pathway and upregulating survivin expression [26]. Pemetrexed may block these receptors interfering with the original ligand which could promote the signaling cascade mentioned above. In addition, as another folate receptor, reduced folate carrier is downregulated in cell lines which developed pemetrexed resistance; a negative feedback mechanism could be speculated on FR-α due to pemetrexed treatment suppressing ERK-signaling pathway, resulting in reduced survivin expression [27].

Even though treatment with the anti-VEGF bevacizumab reduces intracellular survivin concentrations in cancer cells and endothelial cells [28], our data suggest that the extracellular, circulating survivin protein does not change due to platinum plus bevacizumab therapy.

To evaluate whether circulating survivin protein can predict the outcome of advanced stage lung cancer treated with platinum-based chemotherapy, we registered the progression-free and overall survival of our study subjects during a more than three-year follow up period. Although several previous studies confirmed the connection between higher survivin levels and unfavorable outcomes of non-small cell lung cancer and other types of tumors, we did not find correlation between pre-treatment survivin concentrations and the survival parameters of patients.

There are only limited data available about the prognostic or predictive values of survivin in the case of small cell lung cancer. Based on the results of Chen et al. [29] elevated survivin expression proved to be an independent predictor of poor survival outcomes. In contrast, others reported that the nuclear survivin labeling index in the tumor tissue did not correlate with survival [30]. In our study there was a tendency that higher pre-treatment survivin concentrations in SCLC patients were associated with shorter progression-free and overall survival.

The discrepancy between our observations and the results of former investigations can be explained by the different methods of measuring survivin levels. Those studies which supported the role of survivin as a biomarker of lung cancer assessed protein expression by immunohistochemistry on tumor tissue specimens, or measured mRNA levels by PCR in blood samples or in circulating tumor cells. These are reliable methods to examine survivin expression, but their availability is limited, and they require invasive tissue sampling procedures. The assessment of blood survivin protein concentrations can be an option for the non-invasive monitoring of survivin signaling and its dynamics in lung cancer. Regarding other types of malignant diseases, there are contradictory results from studies assessing the diagnostic value of circulating survivin protein levels [31–34]. In non-small cell lung cancer, previous studies could not confirm that survivin protein measured by ELISA in blood samples is a useful biomarker [19, 20].

Several factors can explain our findings. Khan et al. [35, 36] described the process of the exosomal transport of survivin protein from tumor cells to the extracellular space, where survivin creates an extracellular pool. In the tumor microenvironment survivin containing exosomes take part in a dynamic communication network of tumor cells and surrounding stromal and immune cells, and contribute to the adaptation, propagation and spreading of cancer cells. The exosomal release could be influenced by different exogenous factors affecting the cancer cell, which can also change the quantity of the protein in the circulation. Furthermore, systemic inflammatory reaction and endocrine changes related to the advanced malignant disease, and other drugs taken by patient can influence plasma protein levels. In addition, cytostatic chemotherapy and tumor lysis can also significantly affect these features.

In conclusion, we demonstrated a relationship between circulating survivin protein concentrations and the tumor stage as well as the number of metastatic organ sites, which supports previous findings on the role of survivin in tumor spreading. We also revealed a relationship between histological types of lung cancer and survivin concentrations, and a change in survivin levels due to platinum plus pemetrexed chemotherapy. However, to estimate the real clinical implication of our findings, we need more information about the factors which affect the quantity and functioning of extracellular survivin protein *in vivo*.

## Data Availability

The original contributions presented in the study are included in the article/Supplementary Material, further inquiries can be directed to the corresponding author.

## References

[1] Ambrosini G Adida C Altieri DC. A Novel Anti-apoptosis Gene, Survivin, Expressed in Cancer and Lymphoma. Nat Med (1997) 3(8):917–21. 10.1038/nm0897-917 9256286

[2] Altieri DC. Survivin - the Inconvenient IAP. Semin Cel Dev Biol (2005) 39:91–6. 10.1016/j.semcdb.2014.12.007 PMC4410054 25591986

[3] Uren AG Wong L Pakusch M Fowler KJ Burrows FJ Vaux DL Survivin and the Inner Centromere Protein INCENP Show Similar Cell-Cycle Localization and Gene Knockout Phenotype. Curr Biol (2000) 10(21):1319–28. 10.1016/s0960-9822(00)00769-7 11084331

[4] Mita AC Mita MM Nawrocki ST Giles FJ. Survivin: Key Regulator of Mitosis and Apoptosis and Novel Target for Cancer Therapeutics. Clin Cancer Res (2008) 14(16):5000–5. 10.1158/1078-0432.ccr-08-0746 18698017

[5] Kanwar JR Kamalapuram SK Kanwar RK. Targeting Survivin in Cancer: the Cell-Signalling Perspective. Drug Discov Today (2011) 16(11-12):485–94. 10.1016/j.drudis.2011.04.001 21511051

[6] Tamm I Wang Y Sausville E Scudiero DA Vigna N Oltersdorf T IAP-family Protein Survivin Inhibits Caspase Activity and Apoptosis Induced by Fas (CD95), Bax, Caspases, and Anticancer Drugs. Cancer Res (1998) 58(23):5315–20. 9850056

[7] Jaiswal PK Goel A Mittal RD. Survivin: A Molecular Biomarker in Cancer. Indian J Med Res (2015) 141(4):389–97. 10.4103/0971-5916.159250 26112839 PMC4510718

[8] McKenzie JA Liu T Goodson AG Grossman D. Survivin Enhances Motility of Melanoma Cells by Supporting Akt Activation and α5 Integrin Upregulation. Cancer Res (2010) 70(20):7927–37. 10.1158/0008-5472.can-10-0194 20807805 PMC2955769

[9] Fernández JG Rodriguez DA Valenzuela M Calderon C Urzua U Munroe D Survivin Expression Promotes VEGF-Induced Tumor Angiogenesis via PI3K/Akt Enhanced β-catenin/Tcf-Lef Dependent Transcription. Mol Cancer (2014) 9(13):209. 10.1186/1476-4598-13-209 PMC4177250 25204429

[10] Duan L Hu X Jin Y Liu R You Q. Survivin Protein Expression Is Involved in the Progression of Non-small Cell Lung Cancer in Asians: a Meta-Analysis. BMC Cancer (2016) 16:276. 10.1186/s12885-016-2304-3 27090386 PMC4836165

[11] Krepela E Dankova P Moravcikova E Krepelova A Prochazka J Cermak J Increased Expression of Inhibitor of Apoptosis Proteins, Survivin and XIAP, in Non-small Cell Lung Carcinoma. Int J Oncol (2009) 35(6):1449–62. 10.3892/ijo_00000464 19885569

[12] Kapellos G Polonifi K Farmakis D Spartalis E Tomos P Aessopos A Overexpression of Survivin Levels in Circulation and Tissue Samples of Lung Cancer Patients. Anticancer Res (2013) 33(8):3475–80. 23898122

[13] Yie S-m. Lou B Ye S-r. He X Cao M Xie K Clinical Significance of Detecting Survivin-Expressing Circulating Cancer Cells in Patients with Non-small Cell Lung Cancer. Lung Cancer (2009) 63(2):284–90. 10.1016/j.lungcan.2008.05.024 18606477

[14] Hirano H Maeda H Yamaguchi T Yokota S Mori M Sakoda S. Survivin Expression in Lung Cancer: Association with Smoking, Histological Types and Pathological Stages. Oncol Lett (2015) 10(3):1456–62. 10.3892/ol.2015.3374 26622690 PMC4533747

[15] Porebska I Kosacka M Sobanska E Wyrodek E Jankowska R. Comparative Expression of Apoptotic Markers in Lung Adenocarcinoma and Squamous Cell Carcinoma. Adv Exp Med Biol (2015) 873:101–7. 10.1007/5584_2015_121 26022894

[16] Sun P-L Jin Y Kim H Seo AN Jheon S Lee C-T Survivin Expression Is an Independent Poor Prognostic Marker in Lung Adenocarcinoma but Not in Squamous Cell Carcinoma. Virchows Arch (2013) 463(3):427–36. 10.1007/s00428-013-1462-9 23907568

[17] Tang X-P Li J Yu L-C Chen Y-C Shi S-B Zhu L-R Clinical Significance of Survivin and VEGF mRNA Detection in the Cell Fraction of the Peripheral Blood in Non-small Cell Lung Cancer Patients before and after Surgery. Lung Cancer (2013) 81(2):273–9. 10.1016/j.lungcan.2013.05.005 23756092

[18] Du Y-J Li J Zhu W-F Wu Y Tang X-P Wang Y Survivin mRNA-Circulating Tumor Cells Predict Treatment Efficacy of Chemotherapy and Survival for Advanced Non-small Cell Lung Cancer Patients. Tumor Biol (2014) 35(5):4499–507. 10.1007/s13277-013-1592-3 24390669

[19] Fawzy A Gaafar R Kasem F Ali SS Elshafei M Eldeib M. Importance of Serum Levels of Angiopoietin-2 and Survivin Biomarkers in Non-small Cell Lung Cancer. J Egypt Natl Cancer Inst (2012) 24(1):41–5. 10.1016/j.jnci.2011.12.006 23587231

[20] Naumnik W Nilklinska W Ossolinska M Chyczewska E. Serum Levels of HMGB1, Survivin, and VEGF in Patients with Advanced Non-small Cell Lung Cancer during Chemotherapy. Folia Histochem Cytobiol (2009) 47(4):703–9. 10.2478/v10042-009-0024-0 20430742

[21] Eberhardt WE Mitchell A Crowley J Kondo H Kim YT Turrisi A The IASLC Lung Cancer Staging Project: Proposals for the Revision of the M Descriptors in the Forthcoming Eighth Edition of the TNM Classification of Lung Cancer. J Thorac Oncol (2015) 10(11):1515–22. 10.1097/JTO.0000000000000673 26536193

[22] Oh Y Taylor S Bekele BN Debnam JM Allen PK Suki D Number of Metastatic Sites Is a Strong Predictor of Survival in Patients with Nonsmall Cell Lung Cancer with or without Brain Metastases. Cancer (2009) 115(13):2930–8. 10.1002/cncr.24333 19441110

[23] Amini A Li R Liu A Ray P Maniyedath A Huntzinger C Number of Metastatic Organ Sites and Survival by Molecular Profile in Lung Adenocarcinoma. Int J Radiat Oncology (2019) 104(5):1191–2. 10.1016/j.ijrobp.2019.05.049

[24] Wang J Huang C Wei X-y. Qi D-l. Gong L-q. Mu H-y. Changes of Activated Circulating Endothelial Cells and Survivin in Patients with Non-small Cell Lung Cancer after Antiangiogenesis Therapy. Chin Med J (2008) 121(22):2234–40. 10.1097/00029330-200811020-00005 19080323

[25] Derin D Soydinç HO Guney N Tas F Çamlıca H Duranyıldız D Serum Levels of Apoptosis Biomarkers, Survivin and TNF-Alpha in Nonsmall Cell Lung Cancer. Lung Cancer (2008) 59(2):240–5. 10.1016/j.lungcan.2007.08.005 17875341

[26] Zhang J Li Y Wang L Zhang Y Zhang Q Liu J. Folate Receptor Alpha Promotes Endometrial Carcinoma Cell Proliferation and Inhibits Apoptosis by Regulating the ERK Signaling Pathway. Int J Clin Exp Med (2019) 12(7):8791–8.

[27] Liang J Lu T Chen Z Zhan C Wang Q. Mechanisms of Resistance to Pemetrexed in Non-small Cell Lung Cancer. Transl Lung Cancer Res (2019) 8(6):1107–18. 10.21037/tlcr.2019.10.14 32010588 PMC6976363

[28] Xiong Y-Q Sun H-C Zhu X-D Zhang W Zhuang P-Y Zhang J-B Bevacizumab Enhances Chemosensitivity of Hepatocellular Carcinoma to Adriamycin Related to Inhibition of Survivin Expression. J Cancer Res Clin Oncol (2011) 137(3):505–12. 10.1007/s00432-010-0914-8 20490863 PMC11828078

[29] Chen P Zhu J Liu DY Li HY Xu N Hou M. Over-expression of Survivin and VEGF in Small-Cell Lung Cancer May Predict the Poorer Prognosis. Med Oncol (2014) 31(1):775. 10.1007/s12032-013-0775-5 24338338

[30] Yano Y Otsuka T Hirano H Uenami T Satomi A Kuroyama M Nuclear Survivin Expression in Small Cell Lung Cancer. Anticancer Res (2015) 35(5):2935–9. 25964579

[31] Dong H Qian D Wang Y Meng L Chen D Ji X Survivin Expression and Serum Levels in Pancreatic Cancer. World J Surg Oncol (2015) 13:189. 10.1186/s12957-015-0605-7 26016480 PMC4469100

[32] Guney N Soydine HO Derin D Camlica H Duranyildiz D Yasasever V Serum and Urine Survivin Levels in Breast Cancer. Mo (2006) 23(3):335–40. 10.1385/mo:23:3:335 17018890

[33] Tas F Duranyildiz D Argon A Oguz H Camlica H Yasasever V Serum Bcl-2 and Survivin Levels in Melanoma. Melanoma Res (2004) 14(6):543–6. 10.1097/00008390-200412000-00017 15577328

[34] Sohair AH Naema Z Olfat H. Determination of MMP3 and Survivin as Non-invasive Circulating Markers in Patients of Urinary Bladder Cancer. Biohealth Sci Bull (2009) 1(1):29–37.

[35] Khan S Jutzy JMS Aspe JR McGregor DW Neidigh JW Wall NR. Survivin Is Released from Cancer Cells via Exosomes. Apoptosis (2011) 16(1):1–12. 10.1007/s10495-010-0534-4 20717727 PMC3174681

[36] Khan S Aspe JR Asumen MG Almaguel F Odumosu O Acevedo-Martinez S Extracellular, Cell-Permeable Survivin Inhibits Apoptosis while Promoting Proliferative and Metastatic Potential. Br J Cancer (2009) 100:1073–86. 10.1038/sj.bjc.6604978 19293795 PMC2669990

